# Influential Serum Kinases (Non-sFlt-1) and Phosphatases in Preeclampsia—Systematic Review and Metanalysis

**DOI:** 10.3390/ijms241612842

**Published:** 2023-08-16

**Authors:** Karla Cecilia Marrufo-Gallegos, Jose Rafael Villafán-Bernal, Salvador Espino-y-Sosa, Guadalupe Estrada-Gutierrez, Iris Paola Guzmán-Guzmán, Raigam Jafet Martinez-Portilla, Johnatan Torres-Torres

**Affiliations:** 1 Obstetrics and Gynecology Department, Hospital General de Mexico, Mexico City 06720, Mexico; karlacmarrufo@gmail.com; 2 Immunogenomics and Metabolic Diseases, Instituto Nacional de Medicina Genomica, Mexico City 14610, Mexico; joravibe@hotmail.com; 3 Clinical Research Branch, Instituto Nacional de Perinatologia, Mexico City 11000, Mexico; salvadorespino@gmail.com (S.E.-y.-S.); gpestrad@gmail.com (G.E.-G.); raifet@hotmail.com (R.J.M.-P.); 4 Centro de Investigacion en Ciencias de la Salud, Universidad Anahuac, Mexico City 52786, Mexico; 5 American British Cowdray Medical Center IAP, Ob/Gyn Department, Mexico City 01120, Mexico; 6 Faculty of Chemical-Biological Sciences, Universidad Autónoma de Guerrero, Chilpancingo 39030, Mexico; pao_nkiller@yahoo.com.mx

**Keywords:** biomarkers, preeclampsia, serum kinases, sTIE2, c-MET, CK

## Abstract

The early identification of women with an increased risk of preeclampsia (PE) is desirable, but apart from soluble fms-like tyrosine kinase-1 (sFlt-1), few biomarkers have previously been identified as relevant for predicting preeclampsia. Since kinases and phosphatases regulate critical biological processes and previous evidence suggests a potential role of these molecules in preeclampsia, we performed this systematic review and metanalysis. The objective was to determine if there are kinases and phosphatases whose serum levels are different between women with and without PE, being relevant biomarkers of PE. We followed the recommendations of Cochrane and the Preferred Reported Items for Systematic Reviews and Metanalysis (PRISMA) to perform this study. The MESH terms preeclampsia, kinases, phosphatases, angiopoietins, soluble tyrosine protein kinase receptor (sTIE2), and cellular-mesenchymal-epithelial transition factor (c-MET) were combined to find relevant articles in the PubMed, PROSPERO, and Cochrane databases. Then, a qualitative and quantitative analysis was performed in R Studio software. From 580 abstracts identified, 37 were included in the final analysis, which comprised 24,211 pregnant women (2879 with PE and 21,332 women without PE [HP]. The pooled analysis showed that serum creatine kinase (CK) (SMD: 2.43, CI 95% 0.25–4.62) was significantly higher in PE, whereas sTIE2 and anti-angiogenic factor soluble c-Met (sMet)were significantly lower in PE than in HP (SMD: −0.23, CI95% −0.37 to −0.09; and SMD:0.24, CI95% 0.01–0.47, respectively). Adenosine monophosphate-activated protein kinase (AMPK), angiopoietin-1 (ANG-1), angiopoietin-2 (ANG-2), the ratio angiopoietin-1/angiopoietin-2, acid phosphatase, and alkaline phosphatase were not different between women with PE and HP. In summary CK, sTIE2, and c-MET are relevant biomarkers of PE. It is desirable to incorporate them into current models for PE prediction to evaluate their utility as biomarkers.

## 1. Introduction

Preeclampsia (PE) is a multisystemic syndrome affecting 3–5% of all pregnant women, and is characterized by new-onset hypertension associated with organ dysfunction after 20 weeks of gestation, which remains a significant cause of maternal morbidity and mortality [1,2].

Although the etiology of preeclampsia remains incompletely uncovered, some maternal and placental factors are involved in its pathogenesis [3,4]. Under normal conditions, the syncytiotrophoblast releases molecules like vascular endothelial growth factor (VEGF), placental growth factor (PIGF), transforming growth factor-beta (TGF-β), and insulin-like growth factor-1 (IGF-1) that regulate vascular function and help to maintain adequate blood flow to the placenta [4,5]. However, factors inducing oxidative stress, endothelial dysfunction, and inflammation can cause damage to the syncytiotrophoblast, causing a disbalance in the release of growth factors, cytokines, and their soluble forms, including soluble placental growth factor 1 (sFlt-1), VEGF, and PIGF [6]. Consequently, there is a reduction in new blood vessel formation, abnormal placentation, and increased blood pressure, resulting in preeclampsia [7].

sFlt-1 is a circulating antiangiogenic protein that binds to VEGF and PlGF. This interaction disrupts the VEGF pathway, leading to disturbances in endothelial and cellular homeostasis [8]. Preeclampsia is characterized by an imbalance between pro-angiogenic (VEGF or PlGF) and antiangiogenic (sFlt-1) factors in the placenta, resulting in reduced blood flow [9,10]. In pregnant women with preeclampsia, circulating serum levels of sFlt-1 are increased, while PlGF serum concentrations are decreased. The sFlt-1/PlGF ratio is utilized to assess this imbalance and assist in the prediction of preeclampsia [9,10].

Although sFlt-1 is a well-recognized biomarker of PE [9,10,11], we still need to understand the pathophysiology of the disease fully and know all of the signaling pathways and molecular mechanisms involved [7].

Preclinical studies have shown that kinases and phosphatases are important regulators of angiogenesis, vascular stabilization, and endothelial function [12,13,14]. However, while the utility of sFlt-1 as a crucial kinase in preeclampsia has been demonstrated [15,16], it remains unclear whether other serum kinases and phosphatases, routinely measured in patients, could serve as promising biomarkers of the disease, or are implicated in its pathogenesis [17,18].

As a consequence, identifying emerging biomarkers could help us to improve our understanding of the disease’s pathophysiology and the performance of the current models of preeclampsia prediction that already include sFlt-1 and PIGF, or to monitor the efficacy of prophylactic interventions [17,19]. Since systematic reviews and meta-analyses are potent methods to combine and analyze all of the data available in the literature, their use is highly demanded to summarize the existing evidence of new biomarkers for diverse pathologies such as preeclampsia [20,21].

This study aims to identify kinases and phosphatases whose serum levels are different between women, with and without PE, being relevant biomarkers in PE.

## 2. Materials and Methods

### 2.1. Protocol Registration

This study was registered at the prospective international register of systematic reviews (PROSPERO: CDR439182), but no approval from the ethics committee was required to perform this systematic review and meta-analysis.

### 2.2. Information Sources and Search Strategy

A search in the PubMed, Cochrane Library, and PROSPERO databases was performed, limited to humans, in order to find relevant papers related to our objective, including the following keywords: preeclampsia, kinases, phosphatases, angiopoietins, sTIE2, and cMET. The first search was run on March 2023 and updated on 1 May 2023. We strictly adhered to recommendations of the Preferred Reporting Items for Systematic Reviews and Meta-Analyses (PRISMA) guidelines for observational studies.

### 2.3. Eligibility Criteria

Observational studies that reported the serum levels of any kinase or phosphatase (excluding s-FLT1) in at least two groups (preeclampsia versus non-preeclampsia pregnancy) were eligible to be included in the present study. Studies were excluded if not any kinase, phosphatase, or any ratio was estimated from these biomarkers.

### 2.4. Study Selection

Two independent researchers (K.M. and J.R.V.B.) reviewed the abstracts, and were blinded to the authorship, authors’ affiliations, and study results. Data extraction was performed using a standardized form, including study characteristics (author, year, country, study design), participant characteristics, details of serum kinase and phosphatase measurements, and outcome measures. Any discrepancies were resolved through discussion and consensus. If the papers contained information of interest, the full texts were obtained to extract the information of interest. If serum values of a biomarker of interest were only available in graphs, we employed the R software package “digitize” to estimate the levels of such biomarkers accurately. If any disagreement existed between researchers, a third or fourth investigator resolved it. When authors did not provide the interest biomarker’s mean and SD, they were contacted via e-mail. The details of the search syntaxes are presented in Supplementary Materials Table S1.

### 2.5. Assessment of Risk of Bias

The Joanna Briggs Risk of Bias Case Control Tool was used to evaluate the quality of observational studies by two independent reviewers (J.T.T. and I.P.G.G.). The third and fourth evaluators resolved any reviewer disagreements (R.J.M.P. and S.E.S.). The quality of the studies was judged based on three dimensions: the selection of the study groups, the comparability of the groups, and the ascertainment of the exposure.

### 2.6. Data Collection and Analysis

The data of interest were collected on datasheet templates that included the following information: author, year, country where the study was conducted, the kinase or phosphatase measured, inclusion and exclusion criteria, the total number of patients and by group, the trimester of biomarker measurement, and the preeclampsia type. In addition, we obtained the means and standard deviations of serum biomarkers measured.

The serum levels of each biomarker measured in at least two studies were pooled in the meta-analysis, expressing the effect size as a standardized mean difference (SMD) using random-effect model (REM) weighting by inverse of variance, and expressed graphically by means of forest plots. The heterogeneity between studies was calculated through the τ^2^, Cochran’s Q, and I^2^ statistics. If more than five studies were found for an effect size, a Baujat analysis was performed to evaluate the heterogeneity contribution of each study to the overall effect size. A funnel plot was constructed to visually detect bias and systematic heterogeneity. A subgroup analysis was executed to detect differences in biomarkers when enough studies existed to detect differences in SMD by the trimester of gestation.

The statistical analysis was run using the meta, metafor, and metasens packages in R studio v4.2.1 (The R Foundation for Statistical Computing, Indianapolis, IN, USA).

## 3. Results and Discussion

### 3.1. Study Selection and Study Characteristics

**Figure 1:**
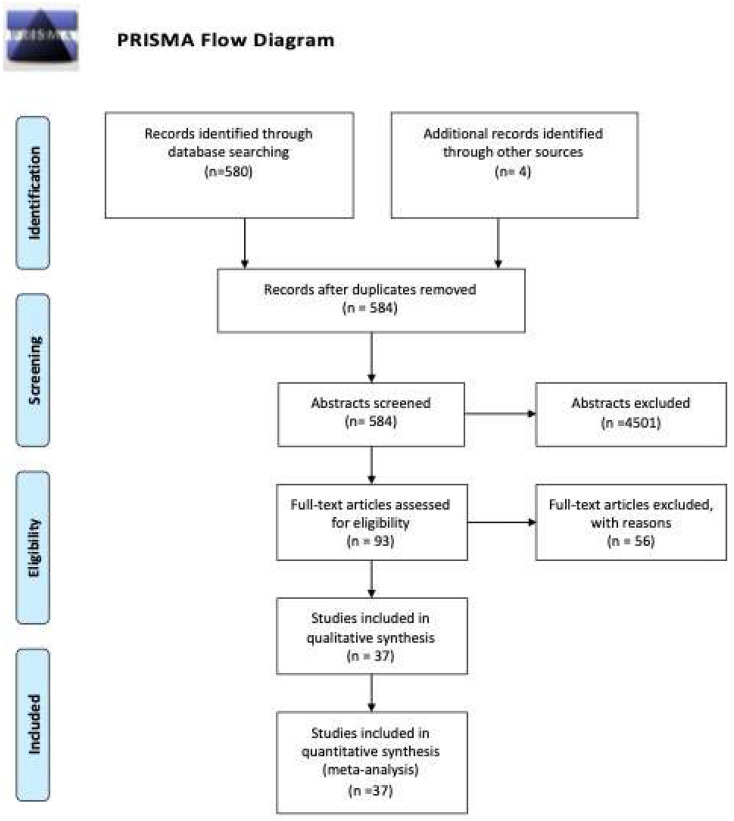
PRISMA flow diagram.

We identified a total of 595 studies through database searching, and 3 manually. After removing the duplicates, 584 abstracts were screened, and 93 studies were eligible for full-text review. Thirty-seven studies were retained for systematic review and meta-analysis. Studies were excluded if they were not case-control or cohort studies, did not provide sufficient data to calculate effect sizes, were review articles, editorials, case reports, or conference abstracts. Supplementary Materials Table S2 contains the reasons for excluding 56 studies. The PRISMA flow diagram is presented in Figure 1. The main characteristics of the included studies are shown in Table 1.

### 3.2. Risk of Bias in the Included Studies

The results of the risk of bias evaluation using the Joanna Briggs Risk of Bias Case Control Tool are shown in Table 2. Most of studies had a moderate risk of bias due to some limitations in the methodology and reporting.

The most common sources of bias were related to the selection of controls, the ascertainment of exposure, and the comparability of cases and controls. Some studies did not adequately match cases and controls, and the criteria for selecting controls were unclear. Additionally, there was variation in how the exposure to serum kinases and phosphatases was measured, which could lead to misclassification of exposure. The outcome assessment showed a low risk of bias in most studies, as the diagnosis of preeclampsia was generally based on established criteria. However, the blinding of outcome assessments were not consistently reported, and some studies lacked blinding, which may introduce bias. The handling of confounding factors and statistical analysis were generally well-addressed in the included studies, with appropriate adjustments for confounders. Withdrawals and dropouts were also adequately addressed in most of the studies.

Based on the assessment of the risk of bias using the Joanna Briggs Risk of Bias Case Control Tool, the included studies demonstrated an overall moderate risk of bias.

### 3.3. Synthesis of Results

In total, 37 studies were included in this meta-analysis comprising 24,211 pregnant women (2879 with preeclampsia and 21,332 controls). Seven studies were performed in early PE, five in late PE, and one in term PE, whereas twenty-nine studies did not specify the PE subtype.

#### 3.3.1. Kinases Significantly Related to Preeclampsia

**Figure 2:**
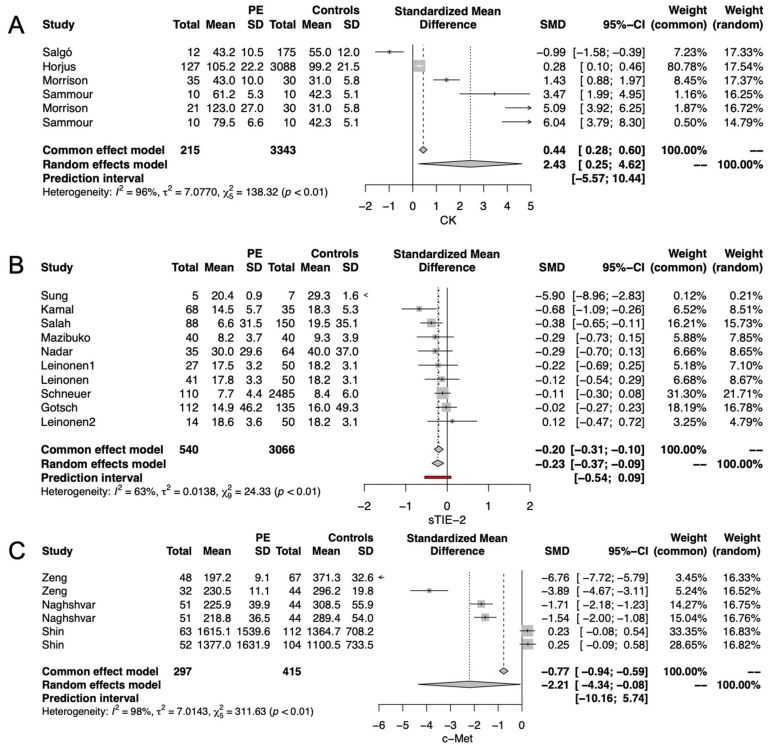
Kinases significantly related to preeclampsia; (**A**) serum creatine kinase (CK); (**B**) soluble tyrosine-protein kinase receptor (sTIE2); (**C**) serum cellular- mesenchymal-epithelial transition factor (c-MET).

The pooled analysis of six studies demonstrated that serum creatine kinase (CK) was significantly higher in PE than in healthy pregnancies (HP) (SMD:2.43, CI 95% 0.25–4.62, *p* < 0.01) (Figure 2A). On the contrary, the metanalysis of eight studies proved that the soluble tyrosine protein kinase receptor (sTIE2) was significantly lower in the serum of PE than in HP (SMD: −0.23, CI 95% −0.37 to −0.09, *p* < 0.001) (Figure 2B). Moreover, the pooled results of three studies revealed lower c-MET serum levels in PE than in HP (SMD: −2.21, CI 95% −4.34 to −0.08, *p* < 0.001) (Figure 2C).

#### 3.3.2. Kinases Non-Significantly Related to Preeclampsia

We found no significant differences in serum levels of AMPK, angiopoietin-1, angiopoietin-2, and the ratio Ang1/Ang2 between PE and HP (Supplementary Materials Figure S1).

#### 3.3.3. Phosphatases and Preeclampsia

Alkaline phosphatase (ALP), acid phosphatase, and heat-stable alkaline phosphatase were not significantly related to preeclampsia (Supplementary Materials Figure S2).

#### 3.3.4. Meta-Regression and Publication Bias

To explain the high I^2^ in CK and c-MET and the moderate I^2^ in the sTie2 estimates, we performed meta-regressions that introduced as covariates gestational age at measurement, maternal age, and pregestational BMI when available. The results indicate that maternal age explained 71.7% of heterogeneity in CK SMD (*p* = 0.0240) (Table 3). In comparison, the factor explaining the high I^2^ of c-MET was pregestational BMI, which accounts for 84.72% of the heterogeneity (*p* < 0.0001) (Table 4). None of the covariates explained the heterogeneity in the sTie2 estimates (Table 5). The funnel plot and Copas analysis revealed evidence of publication bias, and reflected heterogeneity among the studies measuring CK, c-MET, and sTie2 (Supplementary Materials Figure S3).

### 3.4. Main Findings

This study allowed us to identify kinases that were distinct from sFLT-1 and altered in women with preeclampsia compared to women with healthy pregnancies. These biomarkers (CK, sTIE2, and sMET) consistently show differences in women with preeclampsia, and are believed to play a plausible biological role in the development of preeclampsia. It is recommended that these biomarkers be tested in current first-trimester models of preeclampsia to assess their potential for improving the prediction of preeclampsia, and their utility in clinical practice for monitoring the effectiveness of prophylactic interventions, such as aspirin. We found no serum-relevant phosphatases in preeclampsia.

### 3.5. Comparison with Existing Literature and Biological Plausibility of the Findings

Despite there being no previous systematic review and metanalysis performed to identify kinases different from s-Flt-1 and phosphatases as emerging biomarkers of preeclampsia, there is biological plausibility of our findings, and our approach allows us to identify serum biomarkers consistently related to preeclampsia, as we discussed. During normal pregnancy, VEGF, PlGF, and angiopoietins (Ang) help to maintain angiogenesis and endothelial health by interacting with their endogenous endothelial receptors including the vascular endothelial growth factor receptor-1 (VEGFR-1) also called FLT1, the vascular endothelial growth factor receptor 2 (VEGFR-2) also called Kinase insert Domain Receptor (KDR), the Tyrosine kinase with immunoglobulin-like and EGF-like domains 1 (TIE1), and the tyrosine kinase receptor TIE2 [59,60]. However, in PE, an excessive placental secretion of sFlt1 inhibits VEGF signaling in the vasculature, resulting in endothelial cell dysfunction that contributes to PE development [61]. Based on the literature, the lower serum levels of sTIE2 in women with PE found in this systematic review and meta-analysis may be related to the decrease in VEGF signaling induced by the elevation of sFLT-1 [54,62]. Usually, VEGF induces the proteolytic cleavage and shedding of Tie2 [62]. However, we hypothesize that the increase in sFLT1, which occurs in preeclampsia, causes a decrease in VEGF, reducing the proteolytic cleavage and shedding of Tie2. In this sense, a previous study by Findley and colleagues found that decreased circulating soluble Tie2 levels in preeclampsia may result from inhibiting vascular endothelial growth factor (VEGF) signaling [54]. Nevertheless, further research is warranted to comprehensively understand sTIE2′s role in regulating angiogenesis and its impact on the pathophysiology of preeclampsia [54,62]. In another way, MET is primarily found in endothelial and epithelial cells, and participates in angiogenesis [63] during the first and second trimesters of pregnancy [64]. Thus, the pooled lower levels of sMET found in women with preeclampsia are plausible, because this may produce an underdeveloped placental vasculature, facilitating the subsequent progression to preeclampsia. Furthermore, future research involving c-MET in preeclampsia should be complemented with the quantification of liver function tests, because this biomarker is associated with liver function and is elevated in HELLP syndrome [44,58].

The findings of this systematic review and meta-analysis let us recognize potential pathways involved in preeclampsia pathogenesis (the pathways TIE-2 and c-MET), whose components, measured in sera/plasma, may be suitable as emerging biomarkers of preeclampsia. TIE-2 and c-MET participate in two independent pathways that modulate common biological processes, including angiogenesis and macrophage infiltration [65]. In another way, the elevation of CK is consistent with previous observations of single studies where CK was higher in mild and severe preeclampsia than in normal pregnancies [31]. Previous studies also found that plasma CK activity measured in early pregnancy is associated with blood pressure during pregnancy, and is related to severe gestational hypertension [31,48]. Although we cannot explain the biological plausibility of this finding, current evidence suggests that CK could be a potential biomarker in preeclampsia.

### 3.6. Clinical Implications

Identifying biomarkers that accurately predict the development and progression of preeclampsia is crucial for timely interventions and improving maternal and fetal outcomes. In this context, this systematic review and meta-analysis that examined influential serum kinases (non-s-Flt-1) has the following significant clinical implications:

I. Identification of Potential Biomarkers: Our findings provide valuable insights into the role of serum kinases (non-s-Flt-1) that are applicable as potential biomarkers for preeclampsia, since they are clinically different between PE and HP.

II. Diagnostic Accuracy: Our results identified CK, sTIE2, and sMET as kinases significantly related to preeclampsia, and placed them as potential biomarkers to improve the performance of current PE prediction models and enable future timely interventions.

III. Prognostic Value: Understanding the prognostic value of influential serum kinases (non-s-Flt-1) and phosphatases in preeclampsia is crucial for predicting the severity and progression of the disease. The systematic review and meta-analysis provide evidence regarding the associations between these biomarkers and adverse maternal and fetal outcomes. This knowledge enables healthcare providers to identify high-risk cases and implement appropriate monitoring and intervention strategies to mitigate potential complications.

IV. Targeted Therapies and Personalized Medicine: Since our findings identified c-MET and sTIE2 as relevant molecules in PE, targeting these molecular pathways should be tested in the future to determine if their modulation may be preventive or therapeutic targets for this disorder. Our findings contribute to the growing field of personalized medicine by identifying influential serum kinases (non-s-Flt-1) that may help to individualize patient care, as well as interventions to improve outcomes and reduce the burden of PE for the mother and the fetus.

### 3.7. Limitations of the Study

This study has several limitations, including the intrinsic characteristics of preeclampsia, a complex disorder influenced by various genetic, environmental, and clinical factors. In addition, through our systematic review and meta-analysis, we cannot adjust for all potential confounding factors that could influence the serum kinases and phosphatases since we do not have the complete data of each study, including liver function tests. Furthermore, the primary studies are heterogeneous in terms of their design, patient populations, methodologies, and outcome measures. While these limitations do not invalidate the findings, they highlight areas for further research and underscore the need for cautious interpretation and consideration of the broader context. Improvements in the selection of controls, standardization of exposure ascertainment, and blinding of outcome assessment would enhance the validity of future research in this area. Further studies with rigorous methodologies are needed to strengthen the evidence on the associations between serum kinases (different from s-Flt-1) or phosphatases and preeclampsia.

## 4. Conclusions

We identified serum CK, sTIE2, and c-MET as potentially relevant biomarkers for preeclampsia. Our findings suggest that the TIE-2 and the c-MET pathways may influence preeclampsia. Therefore, it is crucial to validate these findings through cohort studies to assess their potential for predicting preeclampsia and improve current first-trimester prediction models. It would also be desirable to investigate the impact of modulating the TIE-2 and c-MET pathways for the prevention and treatment of preeclampsia. However, it is important to interpret these findings cautiously due to limitations of the included studies, such as potential biases. Further prospective studies with larger sample sizes and standardized measurement methods are warranted to address these limitations. These studies would validate the identified biomarkers and explore their clinical utility in predicting and managing preeclampsia.

## Figures and Tables

**Table 1:** General characteristics of the included studies.

Author, Year	Country	Biomolecule	Total Number of Patients/PE Group/Control Group	Trimester of Measurement	Preeclampsia Type
Akolekar, 2009 [22]	UK	ANG-2 (angiopoietin-2)	324/116/208	First	Unspecified
Aoba, 1967 [23]	Japan	ALP (Alkaline Phosphatase)	162/11/151	Second	Severe preeclampsia
Aoba, 1967 [23]	Japan	ALP (Alkaline Phosphatase)	162/11/151	Third	Severe preeclampsia
Aoba, 1967 [23]	Japan	HSAP (Heat stable alkaline phos- phatase)	162/11/151	Second	Severe preeclampsia
Aoba, 1967 [23]	Japan	HSAP (Heat stable alkaline phos- phatase)	162/11/151	Third	Severe preeclampsia
Aoba, 1967 [23]	Japan	HLP (Heat-Labile Alkaline Phosphatase)	162/11/151	second	Severe preeclampsia
Aoba, 1967 [23]	Japan	HLP (Heat-Labile Alkaline Phosphatase)	162/11/151	Third	Severe preeclampsia
Bagga, 1969 [24]	India	ALP (Alkaline Phosphatase)	100/45/55	Third	Unspecified
Bolin, 2009 [25]	Sweden	Ang1/Ang2 ratio	62/19/43	First	Unspecified
Bolin, 2009 [25]	Sweden	Ang1/Ang2 ratio	62/19/43	Second	Unspecified
Bolin, 2009 [25]	Sweden	ANG-2 (angiopoietin-2)	62/19/43	First	Unspecified
Bolin, 2009 [25]	Sweden	ANG-2 (angiopoietin-2)	62/19/43	Second	Unspecified
Bolin, 2009 [25]	Sweden	ANG-2 (angiopoietin-2)	62/19/43	Third	Unspecified
Chen, 2021 [26]	China	ALP (Alkaline Phosphatase)	1012/31/981	First, Second and Third	Unspecified
Gotsch, 2008 [27]	USA	(sTie-2)	247/112/135	Second and Third	Mild, severe, early and late preeclampsia
Han, 2012 [28]	Korea	ANG-2 (angiopoietin-2)	45/16/29	Third	Severe preeclampsia
Hirokoshi, 2005 [29]	Japan	ANG-2 (angiopoietin-2)	55/26/29	Second and Third	Mild and severe preeclampsia
Hirokoshi, 2007 [30]	Japan	ANG-2 (angiopoietin-2)	65/36/29	Second and Third	Mild and severe preeclampsia
Horjus, 2019 [31]	Netherlands	Creatine kinase (CK)	3215/127/3088	First and second	Early preeclampsia
Kamal, 2011 [32]	Egypt	ANG-2 (angiopoietin-2)	103/68/35	Not specified	Unspecified
Karakus, 2015 [33]	Germany	Ang1/Ang2 ratio	62/25/37	Third	Unspecified
Karakus, 2015 [33]	Germany	ANG-2 (angiopoietin-2)	51/17/34	Second and Third	Unspecified
Khalil, 2014 [34]	UK	ANG-2 (angiopoietin-2)	106/22/84	First, Second and Third	Preterm preeclampsia and term preeclampsia
Koroglu, 2018 [35]	Finland	Adenosine AMP-activated protein kinase (AMPK)	80/50/30	Third	Mild and severe preeclampsia
Kumar, 2011 [36]	India	sBAP (serum bone alkaline phosphatase)	120/22/98	Second	Unspecified
Leijnse, 2018 [37]	Netherlands	Ang1/Ang2 ratio	57/6/51	First	Late onset preeclampsia
Leijnse, 2018 [37]	Netherlands	ANG-2 (angiopoietin-2)	57/6/51	First	Late onset preeclampsia
Leinonen, 2009 [38]	Finland	Ang1/Ang2 ratio	91/50/41	Second	Mild and severe preeclampsia
Leinonen, 2009 [38]	Finland	Specific tyrosine kinase receptor (sTie2)	108/49/59	Second	Mild and severe preeclampsia
Leinonen, 2009 [38]	Finland	ANG-2 (angiopoietin-2)	108/49/59	Second	Mild and severe preeclampsia
Machado, 2019 [39]	Brazil	Ang1/Ang2 ratio	120/30/90	Second	Unspecified
Machado, 2019 [39]	Brazil	ANG-2 (angiopoietin-2)	120/30/90	Second	Unspecified
Martinez, 2018 [40]	Mexico	ANG-2 (angiopoietin-2)	36/16/20	Second	Early, late and severe preeclampsia
Mazibuko, 2019 [41]	South Africa	Specific tyrosine kinase receptor (sTie2)	40/20/20	Not specified	Unspecified
Morrison, 1971 [42]	USA	Creatine phosphokinase	65/35/30	Third	Severe preeclampsia
Nadar, 2005 [43]	UK	Ang1/Ang2 ratio	99/35/64	Third	Unspecified
Nadar, 2005 [43]	UK	ANG-2 (angiopoietin-2)	99/35/64	Third	Unspecified
Naghshvar, 2013 [44]	Iran	s-Met (soluble mesenchymal-epithelial transition factor)	95/44/51	First and second	Mild, severe, early and late preeclampsia
Nayel, 1982 [45]	Egypt	ALP (Alkaline Phosphatase)	30/20/10	Third	Severe preeclampsia
Puttapitakpong, 2015 [46]	Japan	ANG-2 (angiopoietin-2)	366/25/341	Second	Early preeclampsia
Aref, 2013 [47]	India	ANG-1 (angiopoietin-1) and Soluble Tie-2 receptor (sTie2)	238/150/88	Not specified	Mild, severe, early and late preeclampsia
Salgó, 1989 [48]	Hungary	Alkaline phosphatase, acid phosphatase and creatine kinase	184/172/12	Second and Third	Unspecified
Sammour, 1974 [49]	Egypt	Creatine phospho-kinase	30/20/10	Third	Mild and severe preeclampsia
Sammour, 1975 [50]	Egypt	HSP (Heat-stable alkaline phosphatase)	30/20/10	Third	Unspecified
Schneuer, 2013 [51]	Australia	ANG-2 (angiopoietin-2)	3893/163/3730	First	Early preeclampsia
Shim, 2015 [52]	Korea	Ang1/Ang2 ratio	74/37/37	Second	Mild and severe preeclampsia
Shim, 2015 [52]	Korea	ANG-2 (angiopoietin-2)	74/37/37	Second	Mild and severe preeclampsia
Shin, 2013 [53]	Seoul	sMet	331/115/216	Second and Third	Unspecified
Sung, 2011 [54]	USA	Specific tyrosine kinase receptor (sTie2)	55/24/31	First, Second and Third	Unspecified
Wang, 2011 [55]	China	ANG-2 (Angiopoietin-2)	92/62/30	Not specified	Moderate and severe preeclampsia
Watson, 1965 [56]	Australia	ALP (Alkaline Phosphatase)	28/3/25	Third	Unspecified
Watson, 1965 [56]	Australia	HSP (Heat-stable alkaline phosphatase)	28/3/25	Third	Unspecified

**Table 2:** Evaluation of risk of bias.

Study	Q1	Q2	Q3	Q4	Q5	Q6	Q7	Q8	Q9	Q10	TOTAL
Akolekar et al., 2009 [22]	YES	YES	YES	YES	YES	YES	UNC	UNC	YES	YES	8
Aoba et al., 1967 [23]	NO	NO	UNC	NO	NO	NO	NO	NO	YES	NO	1
Bagga et al., 1969 [24]	YES	NO	NO	NO	NO	NO	NO	NO	UNC	UNC	1
Bolin et al., 2009 [25]	YES	YES	YES	YES	YES	NO	NO	YES	YES	YES	8
Chen et al., 2021 [26]	UNC	YES	YES	YES	YES	YES	YES	YES	YES	YES	9
Gotsch et al., 2008 [27]	YES	YES	YES	YES	YES	UNC	YES	YES	YES	YES	9
Han et al., 2012 [28]	YES	YES	YES	YES	YES	UNC	YES	YES	NO	YES	8
Hirokoshi et al., 2005 [29]	YES	YES	YES	YES	YES	NO	NO	YES	YES	YES	8
Hirokoshi et al., 2007 [30]	YES	YES	YES	YES	YES	NO	NO	YES	YES	YES	8
Horjus et al., 2019 [31]	YES	YES	YES	YES	YES	YES	YES	YES	YES	YES	10
Kamal et al., 2011 [32]	YES	YES	YES	YES	YES	NO	NO	YES	YES	YES	8
Karakus et al., 2015 [33]	YES	YES	YES	YES	YES	NO	NO	YES	YES	YES	8
Khalil et al., 2014 [34]	YES	YES	YES	YES	YES	YES	YES	YES	UNC	YES	9
Koroglu et al., 2018 [35]	YES	YES	YES	YES	YES	NO	NO	YES	YES	YES	8
Kumar et al., 2011 [36]	YES	YES	YES	YES	YES	NO	NO	YES	YES	YES	8
Leinonen et al., 2009 [38]	YES	YES	YES	YES	YES	NO	NO	YES	YES	YES	8
Leijnse et al., 2018 [37]	YES	YES	YES	YES	YES	YES	NO	YES	YES	YES	9
Machado et al., 2019 [39]	YES	YES	YES	YES	YES	NO	NO	YES	YES	YES	8
Martínez et al., 2018 [40]	YES	YES	YES	YES	YES	NO	NO	YES	YES	YES	8
Mazibuko et al., 2019 [41]	YES	UNC	YES	YES	YES	NO	NO	YES	YES	UNC	6
Morrison et al., 1971 [42]	NO	NO	UNC	YES	UNC	NO	NO	UNC	YES	NO	2
Nadar et al., 2005 [43]	YES	YES	YES	YES	YES	UNC	UNC	YES	YES	YES	8
Naghshvar et al., 2013 [44]	YES	YES	YES	YES	YES	NO	NO	YES	YES	YES	8
Nayel et al., 1982 [45]	YES	UNC	UNC	NO	UNC	NO	NO	NO	YES	NO	2
Puttapitakpong et al., 2015 [46]	YES	YES	YES	YES	YES	NO	NO	YES	YES	YES	8
Aref et al., 2013 [47]	YES	YES	YES	YES	YES	NO	NO	YES	YES	YES	8
Salgo et al., 1989 [48]	NO	NO	NO	NO	UNC	NO	NO	NO	UNC	UNC	0
Sammour et al., 1974 [49]	YES	YES	UNC	YES	YES	NO	NO	YES	UNC	YES	6
Sammour et al., 1975 [50]	YES	YES	YES	YES	YES	NO	NO	YES	YES	YES	8
Schneuer et al., 2013 [51]	YES	NO	YES	UNC	UNC	NO	NO	YES	YES	YES	5
Shim et al., 2015 [52]	YES	YES	YES	YES	YES	NO	NO	YES	YES	YES	8
Sung et al., 2011 [54]	YES	YES	YES	YES	YES	NO	NO	YES	YES	YES	8
Wang et al., 2011 [55]	YES	YES	YES	YES	YES	NO	NO	YES	UNC	YES	7
Watson et al., 1965 [56]	NO	NO	YES	UNC	UNC	NO	NO	UNC	YES	UNC	2
Young et al., 1968 [57]	NO	YES	YES	YES	YES	NO	NO	YES	YES	YES	7
Kim et al., 2013 [53]	YES	YES	YES	YES	YES	YES	YES	YES	YES	YES	10
Zeng et al., 2009 [58]	YES	YES	YES	YES	YES	NO	NO	YES	YES	YES	8
Q1. Were the groups comparable other than the presence of disease in cases or the absence of disease in controls? Q2. Were cases and controls matched appropriately? Q3. Were the same criteria used for the identification of cases and controls? Q4. Was exposure measured in a standard, valid, and reliable way? Q5. Was exposure measured in the same way for cases and controls? Q6. Were confounding factors identified? Q7. Were strategies to deal with confounding factors stated? Q8. Were outcomes assessed in a standard, valid, and reliable way for cases and controls? Q9. Was the exposure period of interest long enough to be meaningful? Q10. Was appropriate statistical analysis used?											

Green—Low risk of bias. Yellow—Unclear risk of bias. Red—High risk of bias.

**Table 3:** Meta-regression analysis of heterogeneity modulators in CK.

Covariate/Modulator	Estimate	95% CI		*p*-Value	R2 (%)
Gestational Age	0.01794	−0.0316	0.3903	0.0956	26.59
Maternal Age	−1.4117	−2.6379	−0.1856	0.0240	71.70
Pregestational BMI	-	-	-	-	-

**Table 4:** Meta-regression analysis of heterogeneity modulators in c-MET.

Covariate/Modulator	Estimate	95% CI		*p*-Value	R2 (%)
Gestational Age	−0.2335	−0.4834	0.0163	0.0670	32.63
Maternal Age	0.1996	−0.5839	0.9831	0.6176	0.00
Pregestational BMI	−24.0521	−35.7118	−12.3924	<0.001	84.72

**Table 5:** Meta-regression analysis of heterogeneity modulators in sTie2.

Covariate/Modulator	Estimate	95% CI		*p*-Value	R2 (%)
Gestational Age	−0.0078	−0.0226	0.0069	0.2964	0.000
Maternal Age	−0.0214	−0.0646	0.0217	0.3295	0.000
Pregestational BMI	−0.1046	−0.3795	0.1703	0.9042	0.000

## Data Availability

The data employed for conducting this metanalysis are available if requested to the following e-mails: jvillafan@inmegen.edu.mx; torresmmf@gmail.com.

## Supplementary Materials

The following supporting information can be downloaded at: https://www.mdpi.com/article/10.3390/ijms241612842/s1, https://drive.google.com/drive/folders/13rBqSM_TZTSvTE-w5fzhEjDG4moSKzhW?usp=drive_link. Just send an e-mail to jvillafan@inmegen.edu.mx or torresmmf@gmail.com and a password will be provided.
