# Supplementary material for: Influential Serum Kinases (Non-sFlt-1) and Phosphatases in Preeclampsia—Systematic Review and Metanalysis
Source: Int J Mol Sci. 2023 Aug 16;24(16):12842. doi: 10.3390/ijms241612842 (PMC10454832; doi:10.3390/ijms241612842)
Supplement: Supplementary file 1 [file ijms-24-12842-s001.zip › ijms-2520070-supplementary.pdf]

**Supplementary Table S1.** Details of search syntaxes
Search: **(serum OR plasma) AND (protein kinase OR kinase or kinases OR phosphatase OR sTIE2 OR sMET OR angiopoietin) AND (preeclampsia) NOT sFLT-1** Sort by: **Most Recent**
(("serum"[MeSH Terms] OR "serum"[All Fields] OR "serums"[All Fields] OR "serum s"[All Fields] OR "serumal"[All Fields] OR ("plasma"[MeSH Terms] OR "plasma"[All Fields] OR "plasmas"[All Fields] OR "plasma s"[All Fields])) AND ("protein kinases"[MeSH Terms] OR ("protein"[All Fields] AND "kinases"[All Fields]) OR "protein kinases"[All Fields] OR ("protein"[All Fields] AND "kinase"[All Fields]) OR "protein kinase"[All Fields] OR ("kinase s"[All Fields] OR "phosphotransferases"[MeSH Terms] OR "phosphotransferases"[All Fields] OR "kinase"[All Fields] OR "kinases"[All Fields]) OR ("kinase s"[All Fields] OR "phosphotransferases"[MeSH Terms] OR "phosphotransferases"[All Fields] OR "kinase"[All Fields] OR "kinases"[All Fields]) OR ("phosphatase s"[All Fields] OR "phosphoric monoester hydrolases"[MeSH Terms] OR ("phosphoric"[All Fields] AND "monoester"[All Fields] AND "hydrolases"[All Fields]) OR "phosphoric monoester hydrolases"[All Fields] OR "phosphatase"[All Fields] OR "phosphatases"[All Fields]) OR "sTIE2"[All Fields] OR "sMET"[All Fields] OR ("angiopoietins"[MeSH Terms] OR "angiopoietins"[All Fields] OR "angiopoietin"[All Fields])) AND ("pre eclampsia"[MeSH Terms] OR "pre eclampsia"[All Fields] OR "preeclampsia"[All Fields])) NOT "sFLT-1"[All Fields]
**Translations**<br>**serum:** "serum"[MeSH Terms] OR "serum"[All Fields] OR "serums"[All Fields] OR "serum's"[All Fields] OR "serumal"[All Fields]<br>**plasma:** "plasma"[MeSH Terms] OR "plasma"[All Fields] OR "plasmas"[All Fields] OR "plasma's"[All Fields]<br>**protein kinase:** "protein kinases"[MeSH Terms] OR ("protein"[All Fields] AND "kinases"[All Fields]) OR "protein kinases"[All Fields] OR ("protein"[All Fields] AND "kinase"[All Fields]) OR "protein kinase"[All Fields]<br>**kinase:** "kinase's"[All Fields] OR "phosphotransferases"[MeSH Terms] OR "phosphotransferases"[All Fields] OR "kinase"[All Fields] OR "kinases"[All Fields]<br>**kinases:** "kinase's"[All Fields] OR "phosphotransferases"[MeSH Terms] OR "phosphotransferases"[All Fields] OR "kinase"[All Fields] OR "kinases"[All Fields]<br>**phosphatase:** "phosphatase's"[All Fields] OR "phosphoric monoester hydrolases"[MeSH Terms] OR ("phosphoric"[All Fields] AND "monoester"[All Fields] AND "hydrolases"[All Fields]) OR "phosphoric monoester hydrolases"[All Fields] OR "phosphatase"[All Fields] OR "phosphatases"[All Fields]<br>**angiopoietin:** "angiopoietins"[MeSH Terms] OR "angiopoietins"[All Fields] OR "angiopoietin"[All Fields]<br>**preeclampsia:** "pre-eclampsia"[MeSH Terms] OR "pre-eclampsia"[All Fields] OR "preeclampsia"[All Fields]
https://pubmed.ncbi.nlm.nih.gov/?term=%28serum+OR+plasma%29+AND+%28protein+kinase+OR+kinase+or+kinases+OR+phosphatase%29+AND+%28preeclampsia%29+NOT+sFLT-1&format=abstract&size=200

Supplementary Table S2. Reasons for exclusion

Author	Year	Reason of exclusion
Grimaldi	2022	This is an in vitro study, not performed in human
Liu	2022	This is an in vitro study, not performed in human
Nevalein	2017	This is a meta-analysis
Mbhele	2017	Some patients had HIV
Lekva	2016	This is an in vitro study, not performed in human
Kappou	2015	This is a review of other studies
Verlohren	2014	Is a response to Khalil's 2014 article
Kappou	2014	This is an in vitro study, not performed in human
Vasilopoulou	2014	This is an in vitro study, not performed in human
Robson	2012	This is an in vitro study, not performed in human
Farina	2011	This is an in vitro study, not performed in human

Seval	2008	This is an in vitro study, not performed in human
Puchner	2006	Serum Ang 2 concentration is only reported in IUGR
Korff	2004	This is an in vitro study, not performed in human
Geva	2002	This is an in vitro study, not performed in human
Li	2001	This is an in vitro study, not performed in human
Özgökçe	2023	The ang1/ang2 ratio is not measured
Yang	2023	It is an In vitro study, and the ang1/ang2 ratio are not measured
Morgaan	2023	It is an In vitro study, and the ang1/ang2 ratio are not measured
Abuiessa	2022	It is an In vitro study, and the ang1/ang2 ratio are not measured
Yu	2022	It is an In vitro study, and the ang1/ang2 ratio are not measured
Lee	2020	It is an In vitro study, and the ang1/ang2 ratio are not measured
Tanabe	2020	The ang1/ang2 ratio are not measured
Zhao	2019	This is an in vitro study, not performed in human
Umapathy	2019	The ang1/ang2 ratio are not measured
Kappou	2015	The ang1/ang2 ratio are not measured
Kappou	2014	It is an In vitro study, and the ang1/ang2 ratio are not measured
Aref	2013	The ang1/ang2 ratio are not measured
Seval	2008	This is an in vitro study, not performed in human
Guschmann	2003	This is an in vitro study, not performed in human
Svedas	2003	This is an in vitro study, not performed in human
Geva	2002	This is an in vitro study, not performed in human
Xiao Feng	2001	This is an in vitro study, not performed in human
Jiang	2001	This is an in vitro study, not performed in human
Muñoz	2017	Don't measuring kinases or phosphatases different to sFLT-1 or sEng
Cim	2017	Don't measuring kinases or phosphatases different to sFLT-1 or sEng
Wu	2021	This is an in vitro study, not performed in human
Maynard	2010	Don't measuring kinases or phosphatases different to sFLT-1 or sEng
Moore	2013	Don't measuring kinases or phosphatases different to sFLT-1 or sEng
Ogunleye	2017	This is an in vitro study, not performed in human
Abid	2020	This is an in vitro study, not performed in human
Haller	1989	This is an in vitro study, not performed in human
Rodrigues	2018	This is a systematic review
Zhang	2021	This is an in vitro study, not performed in human
Horjusa	2019	Study not conducted in pregnant women, only in patients with a history of preeclampsia
Oggeé	2010	Don't measuring kinases or phosphatases different to sFLT-1
Palomo	2022	This is an in vitro study, not performed in human
Thakoordeen	2017	Patients had HIV
Jimenez	2003	This is an in vitro study, not performed in human
Khaliq	2018	This is an in vitro study, not performed in human
Liu	2003	This is an in vitro study, not performed in human
Tang	2014	This is an in vitro study, not performed in human
Orozco	2009	This is an in vitro study, not performed in human
Sakowicz	2021	This is an in vitro study, not performed in human
Curzen	1971	Phosphatase values are not reported in preeclampsia, only if the level was normal or abnormal
McCorry	1974	This is an in vitro study, not performed in human

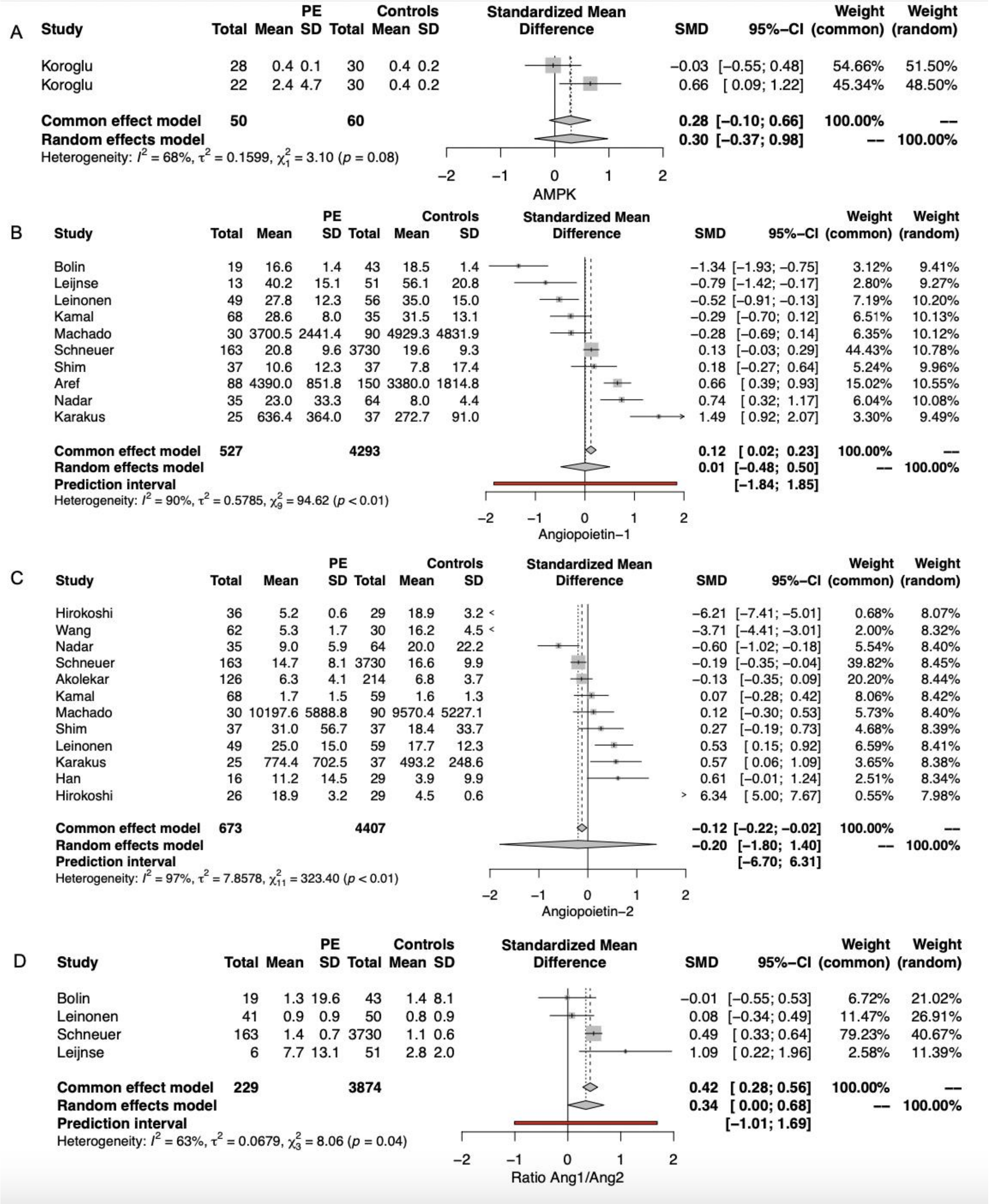

Figure S1. **(A)** Adenosine AMP-activated protein kinase (AMPK); **(B)** angiopoietin-1; **(C)** angiopoietin-2; ratio Ang1/Ang2.

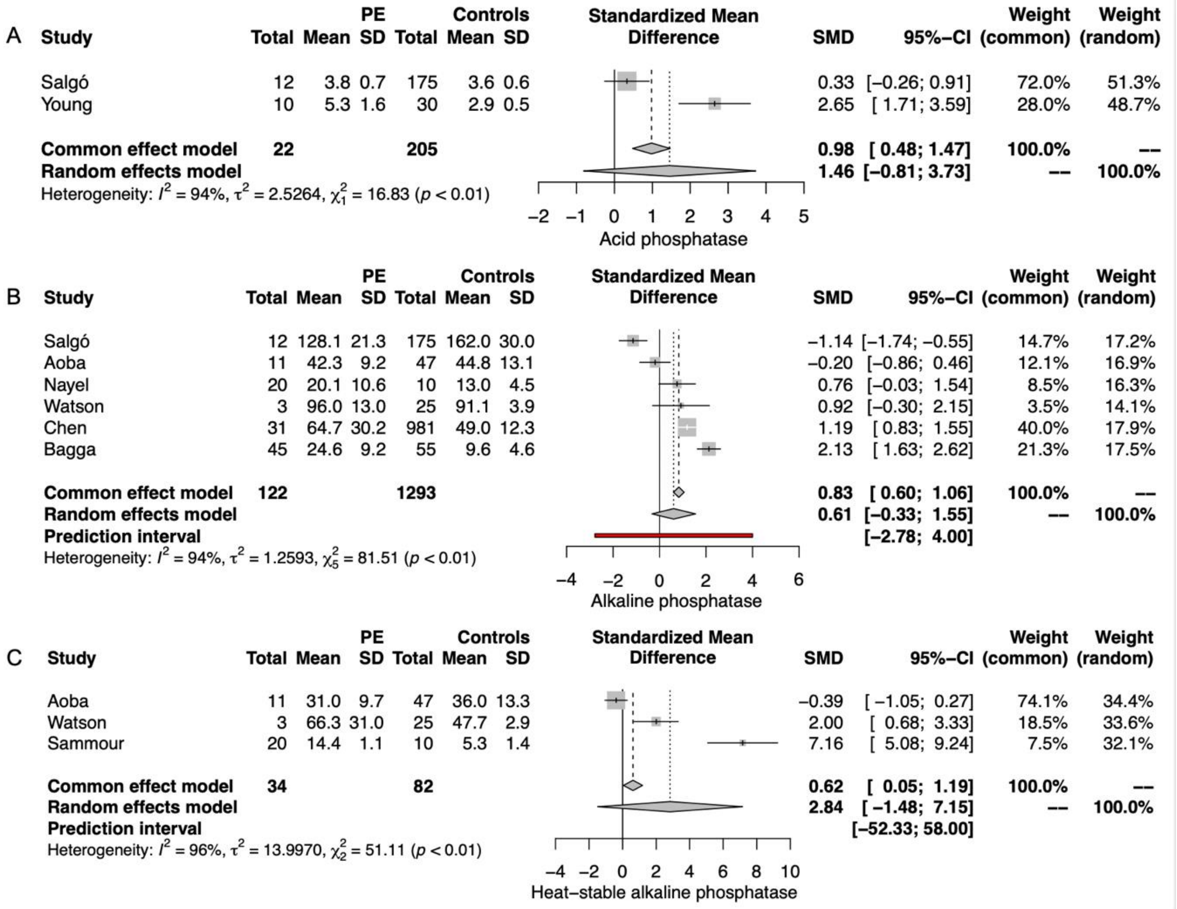

Figure S2. **(A)** Acid phosphatase; **(B)** alkaline phosphatase (ALP); **(C)** heat-stable alkaline phosphatase.

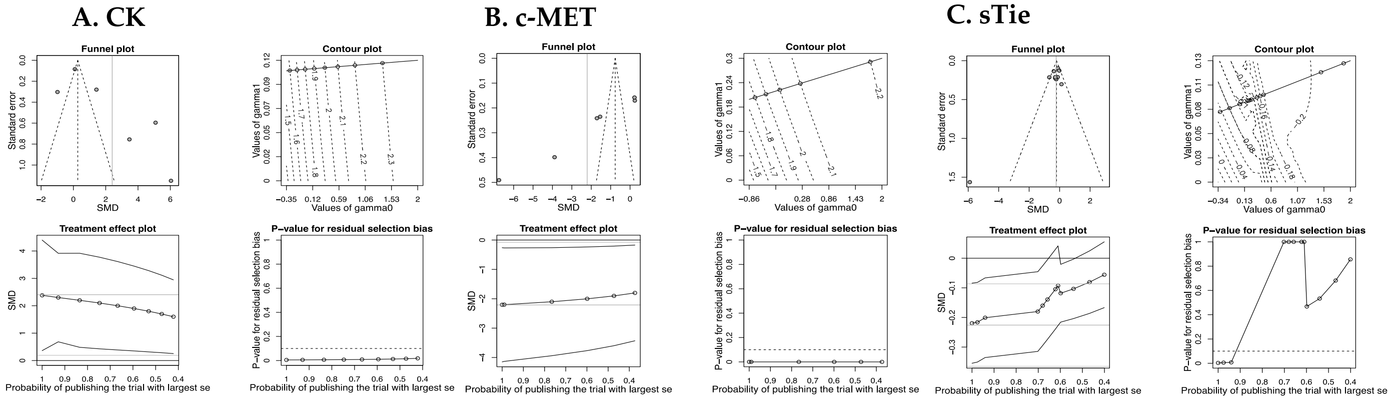

Figure S3. Evaluation of publication bias and study heterogeneity through Funnel plot and Copas analysis. **(A)** serum creatine kinase (CK); **(B)** protein tyrosine kinase Met (c-MET); **(C)** soluble tyrosine-protein kinase (sTIE2).
